# Biofloc Technology for Nile Tilapia Fry: Technical and Economic Feasibility, Solids Control, and Stocking Density

**DOI:** 10.3390/ani15202942

**Published:** 2025-10-10

**Authors:** Raphael de Leão Serafini, Bruno Corrêa da Silva, Haluko Massago, Eduardo da Silva, Adolfo Jatobá

**Affiliations:** 1 Empresa de Pesquisa Agropecuária e Extensão Rural de Santa Catarina (EPAGRI), Rodovia Antônio Heil, Itaipava, Itajaí 88318-112, SC, Brazil; raphaelserafini@epagri.sc.gov.br (R.d.L.S.); brunosilva@epagri.sc.gov.br (B.C.d.S.);; 2 Medicina Veterinária, Faculdade Life Unic Education (Life Unic), R. Sen. Felipe Schmidt, 159, Joinville 89201-440, SC, Brazil; 3 Medicina Veterinária, Centro Universitário Avantis (UNIAVAN)—Balneário Camboriú, Av. Marginal Leste, 3600, Balneário Camboriú 88339-125, SC, Brazil; 4 Aquaculture Laboratory, Catarinense Federal Institute, Campus Araquari (IFC), BR-280 Km 27, Araquari 89245-000, SC, Brazil

**Keywords:** *Oreochromis niloticus*, sex reversal, water quality, zootechnical performance

## Abstract

**Simple Summary:**

Aquaculture is one of the fastest growing food production sectors, but its success depends on developing methods that reduce costs and environmental impacts. This study evaluated the use of the biofloc system, a technique that uses microorganisms and organic matter present in the water to improve water quality and provide natural food for fish. We tested whether this system could support the early growth of Nile tilapia fry under different stocking densities, and whether controlling the amount of solids in the water would improve results. Our findings showed that the biofloc system can partially replace feed, maintain water quality, and support the growth of tilapia fry. These results demonstrate that the system is technically feasible and may reduce production costs in aquaculture.

**Abstract:**

This study evaluated the technical and economic feasibility of the biofloc technology (BFT) system during the fry rearing phase of Nile tilapia (*Oreochromis niloticus*), focusing on suspended solids management, stocking density, and economic performance at a pilot scale. Three trials were conducted. The first assessed the effects of four total suspended solids (TSS) ranges (0–200, 200–400, 400–600, and 600–800 mg·L^−1^) on larval performance and water quality. TSS levels between 200 and 600 mg·L^−1^ promoted improved water quality and zootechnical performance. The second trial tested five stocking densities (2, 4, 6, 8, and 10 larvae·L^−1^), evaluating their impact on water quality, survival, and size uniformity. Higher densities negatively affected survival (R^2^ = 0.84) and final weight (R^2^ = 0.92), while also increasing solids and nitrogenous compounds, thus impairing performance (*p* < 0.05). The third trial monitored six production cycles at pilot scale, evaluating zootechnical parameters, sex reversal efficiency, and economic indicators. All cycles showed survival rates above 85%, sex reversal close to 100%, and positive net margins (18.5 to 41.9%), demonstrating the viability of BFT for commercial fry operations. The results emphasize the importance of controlling suspended solids and stocking density to maintain water quality and optimize larval performance. Furthermore, the system proved economically viable, with good feed conversion rates and profitability, even without water exchange. These findings support BFT as a sustainable and efficient alternative for tilapia fry production, offering significant water savings and promising economic returns when properly managed.

## 1. Introduction

Aquaculture has increasingly established itself as a major global provider of fish, with certain species standing out, such as Nile tilapia (*Oreochromis niloticus*) [1,2]. Over the past ten years, Nile tilapia production has grown steadily, with a 14.36% increase in 2024 compared to the previous year [2]. This growth is attributed to the species’ rising value as an affordable, high-quality protein source, as well as its zootechnical characteristics, including robustness, high adaptability to intensive farming systems, and rapid growth [3].

However, this production growth must be accompanied by sustainable practices and management strategies aimed at reducing environmental impacts and optimizing resource use [4]. Conventional systems that rely on continuous water exchange have proven to be inefficient in terms of production per cubic meter of water used when compared to systems with water reuse [5,6]. Therefore, alternative production systems such as biofloc technology (BFT) have gained attention as a promising and sustainable option, both for advanced stages of cultivation and early life stages such as post-larvae and fry [7,8,9,10,11]. The biofloc system uses organic solids such as uneaten feed and fish excreta, which are transformed by microbial activity into aggregates rich in bacteria, algae, and protozoa [8,9]. These microorganisms form aggregates (bioflocs) that can be consumed by fish, providing protein, lipids, and vitamins to their diet, thereby reducing reliance on conventional feed and enhancing sustainability [7,8]. This reduces the need for external inputs such as feed and improves the utilization of already introduced resources [9]. The system has the potential to enhance productivity, improve feed conversion, and significantly reduce water use [7]. Despite these advantages, BFT presents challenges, including waste management (effluents, suspended solids), maintaining appropriate stocking densities to prevent water quality deterioration, and relatively high initial implementation costs [7,9]. High concentrations of total suspended solids (TSS) can cause skin irritation, fin erosion, blockage of the opercular cavity, inhibition of gas diffusion, excretion of nitrogen compounds, and alterations in ion exchange [11]. Stocking density directly influences production and profitability; however, when high, it demands strict monitoring and continuous maintenance of water quality, as increased density accelerates its deterioration [11]. Moreover, the rearing of early life stages is particularly challenging due to the immature immune system of the fry, requiring a stable and safe environment [12,13], which must also be economically feasible and compatible with management techniques such as sex reversal.

An all-male population is used to prevent reproduction, and hormonal treatment is applied to achieve this [11,12,13]. However, despite the many advantages of BFT, concerns have been raised about the effectiveness of sex reversal in this system, as fish feeding on bioflocs may consume lower amounts of the hormone-treated feed necessary for successful reversal [11]. Nevertheless, previous studies have shown that sex reversal protocols can still be successfully applied within BFT systems [12].

Although studies have addressed BFT and sex reversal management in Nile tilapia, there is limited knowledge about the overall economic viability of this approach—particularly when applied in combination. Therefore, the aim of this study was to evaluate the technical and economic feasibility of using the biofloc technology (BFT) system during the nursery phase of Nile tilapia (*Oreochromis niloticus*) at a pilot scale.

## 2. Materials and Methods

The experiments were conducted using Nile tilapia (*O. niloticus*) larvae obtained from eggs collected during spawning events at the Itajaí Experimental Aquaculture Station (CEPIT), which belongs to the Agricultural Research and Rural Extension Company of Santa Catarina (EPAGRI). The larvae used were approximately three days post-hatch, ensuring an appropriate size for achieving a high sex reversal rate [14]. All procedures adopted in this study followed the guidelines of the Animal Ethics Committee and were approved by CEUA-EPAGRI under protocol numbers 006/2021 and 001/2024.

### 2.1. Experimental Structure

The first two experimental trials were conducted in a controlled environment within the Bioassay Laboratory at CEPIT. Experimental units with a working volume of 100 L were used, each equipped with an individual aeration system and 200 W heaters with thermostats (set at 28 ± 1 °C). A central aeration system using aerotube diffusers was also installed to maintain bacterial flocs in suspension and keep dissolved oxygen levels within the appropriate range for cultivation. Initially, the experimental units were inoculated with previously matured biofloc to reach the desired initial solids concentration and filled with pre-filtered water (50 µm), disinfected with chlorine (10 mg·L^−1^), and dechlorinated through aeration.

### 2.2. Trial 1: Evaluation of Different Total Suspended Solids Management Strategies in Nile Tilapia Fry Rearing in a Biofloc System

The first experiment evaluated the performance of Nile tilapia larvae reared under different total suspended solids (TSS) management strategies in a biofloc system. Each experimental unit was stocked with Nile tilapia larvae at a density of three larvae per liter (average weight of 11.9 mg), totaling 300 animals per tank. A total of 16 experimental units were used, arranged in a completely randomized design with four treatments and four replicates each.

**Figure 1:**
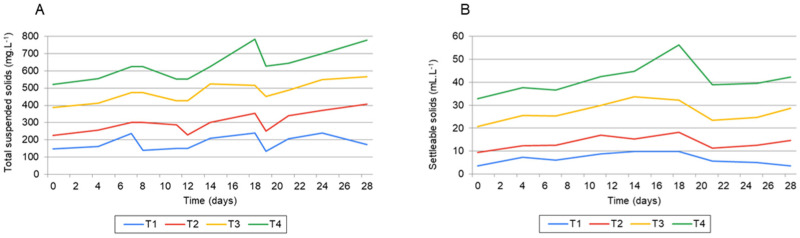
(**A**) Total suspended solids (mg·L^−1^) as per different solids management over time in Nile tilapia hatchery. (**B**) Settleable solids (mL·L^−1^) as per different solids management over time in Nile tilapia hatchery. T1: 0–200 mg·L^−1^; T2: 200–400 mg·L^−1^; T3: 400–600; mg·L^−1^; T4: 600–800 mg·L^−1^.

The experimental treatments consisted of maintaining different TSS concentration ranges throughout the larviculture period: 0–200 mg·L^−1^ (T1); 200–400 mg·L^−1^ (T2); 400–600 mg·L^−1^ (T3); and 600–800 mg·L^−1^ (T4). Figure 1 shows the measured values of total suspended solids (TSS) and settleable solids (SS) throughout the experimental period.

To maintain total suspended solids (TSS) within the predefined levels, a 5000-L stock tank containing juvenile tilapia was used. A clarifier was installed in this tank to keep the TSS concentration close to that of the highest treatment. To obtain the other target concentrations, water from the stock tank was diluted with water from the clarifier’s outlet (which had low TSS levels), enabling the desired TSS ranges to be achieved.

TSS concentrations were measured twice weekly, and when necessary, partial water filtration was performed on the experimental units using a 50-micron BAG filter to reduce TSS. The estimated amount of solids removed was calculated using the following equation:

$$Vf=Vt-\left(\frac{TSSd\times Vt}{TSSa}\right)$$

where *Vf* is the volume to be filtered (L); *Vt* is the tank volume (100 L); *TSSd* is the desired total suspended solids concentration (value in mg·L^−1^ according to the treatments); and *TSSa* is the measured total suspended solids concentration (mg·L^−1^).

At the end of the experiment, the total amount of solids removed per experimental unit was also calculated.

Biometric measurements were taken weekly to adjust feed amounts, according to the feeding management protocol described in Section 2.4. Water quality was monitored throughout the experiment, as detailed in Section 2.5. After 28 days, all experimental units were harvested, and the fish were counted and weighed to obtain performance data, as described in Section 2.6.

### 2.3. Trial 2: Evaluation of Stocking Density in Nile Tilapia Larvae Cultivated in a Biofloc System

The second experiment evaluated the effect of stocking density on the performance of Nile tilapia larvae reared in a biofloc system. The following stocking densities were tested: 2, 4, 6, 8, and 10 larvae per liter.

Larval feeding was carried out as described in Section 2.4. Water quality was monitored throughout the experiment, as outlined in Section 2.5. During this trial, efforts were made to maintain TSS concentrations between 200 and 600 mg·L^−1^. When necessary, TSS levels were controlled as described in Section 2.2.

After 28 days, all experimental units were harvested, and the fish were counted and weighed to obtain performance data, as described in Section 2.6. In addition, all fish from each experimental unit were manually graded into three size classes using a tubular grader, based on the grid openings: P—<7.5 mm; M—7.5 to 9.0 mm; L—>9.0 mm.

From the weight and number of animals in each size class, the average weight and the proportion of each class within the total batch were calculated.

### 2.4. Feeding Management

The larvae were fed a powdered diet containing 52% crude protein and 60 mg kg^−1^ of masculinizing hormone (17-ɑ-methyltestosterone), following the protocol proposed by Guerrero [15]. The feeding frequency was four meals per day throughout the 28-day experimental rearing period.

The daily feed amount was calculated based on a feeding table adapted from Popma & Green [16] for sex reversal in hapas, ranging from 2.5 g of feed per 1000 larvae per day at the beginning of the nursery phase, up to 46 g per 1000 larvae per day by the end of the 28-day period.

### 2.5. Water Quality Monitoring and Management in the Studies

Dissolved oxygen (DO) and temperature were measured daily using a digital oximeter (YSI, model Pro 20), approximately 30 min after the first feeding. Twice a week, analyses of pH (YSI, model Professional Plus), total ammonia nitrogen (TAN), nitrite, volume of settleable solids, total suspended solids, and alkalinity were performed. Nitrate and water hardness were measured at the beginning (day 1), middle (day 14), and end (day 28) of the experiment.

TAN, nitrite, and nitrate analyses were carried out using a microprocessor-based photocolorimeter with colorimetric kits (Alfakit^®^, Florianópolis, Brazil). Ammonia was determined using the indophenol colorimetric method (4500-NH_3_ F) [17], nitrite using the diazotization colorimetric method (4500-NO_2_^−^ B) [17], and nitrate by the brucine method [18]. Alkalinity and hardness were determined using the titration method [17] with a colorimetric kit (Alfakit^®^, Florianópolis, Brazil). The volume of settleable solids (VSS) was measured using an Imhoff cone, and total suspended solids (TSS) were determined following APHA [17] methodology.

During the study, no water renewal was performed in the biofloc system; only evaporative losses were replenished weekly. Twice a day (8:00 a.m. and 5:00 p.m.), sodium bicarbonate (NaHCO_3_) was added to the water in the experimental units to maintain alkalinity between 60 and 100 mg·L^−1^ and pH between 7.5 and 8.0. The amount of sodium bicarbonate added was calculated based on the percentage of feed offered daily (*w*/*w*), ranging from 20 to 30% of the daily feed input. This proportion was adjusted according to pH and alkalinity analyses. Additionally, water salinity during the nursery phase was maintained at 2.0 ± 0.2 ppt using sodium chloride.

### 2.6. Zootechnical Performance

Fish growth was evaluated through weekly biometrics of at least 10% of the animals stocked in each experimental unit. These animals were randomly sampled to obtain the weekly average weight. At the end of the experiment, the fish were weighed and counted to determine the final average weight, specific growth rate, daily weight gain, feed conversion ratio, productivity, and final survival. The following equations were used for these calculations:

$$SGR=100\times \left(\frac{ln\left(Wf\right)-ln\left(Wi\right)}{t}\right)$$

where:

SGR—Specific Growth Rate (%);
Wf—Final average weight (g);
Wi—Initial average weight (g);
t—Culture period (days).

$$DWG\left(g\right)=\frac{\left(Wf-Wi\right)}{t}$$

where:

DWG—Daily Weight Gain (g·day^−1^);
Wf—Final average weight (g);
Wi—Initial average weight (g);
t—Culture period (days).

$$FCR=\frac{TF}{\left(Bf-Bi\right)}$$

where:

FCR—Feed Conversion Ratio;
TF—Total feed supplied (g);
Bf—Final biomass (g);
Bi—Initial biomass (g).

$$Final\;productivity\left(\frac{kg}{m^{3}}\right)=\frac{Bf\left(kg\right)}{V\left(m^{3}\right)}$$

where:

BF—Final biomass (kg);
V—Volume of the experimental unit (m^3^).

$$Survival\;rate\;\left(\%\right)=100\times \frac{N_{f}}{N_{i}}$$

where:

Nf—Final number of fish;
Ni—Initial number of stocked fish.

### 2.7. Statistical Analyses

Water quality data and zootechnical performance data from the solid management trial were subjected to analysis of variance (ANOVA) after testing for data normality (Shapiro–Wilk test) and homogeneity of variances (Levene’s test). When a significant difference among treatments was observed, Tukey’s test was used for mean comparison. Zootechnical performance data from the stocking density trial were analyzed using polynomial regression. The significance level adopted was 0.05. All statistical analyses were performed using R software (R version 4.1.0) [19].

### 2.8. Trial 3: Pilot-Scale Production Monitoring

Throughout two production seasons (2023/2024 and 2024/2025), six nursery cycles of Nile tilapia during the sex reversal phase were monitored. The production units used were 12 m^3^ geomembrane tanks (ø = 4.5 m, average height = 0.76 m), installed inside a 300 m^2^ greenhouse. The greenhouse was covered with 200-micron transparent polyethylene film and 70% shade cloth, and equipped with side curtains to regulate internal temperature. The aeration system consisted of a 7.5 hp radial air compressor connected to a frequency inverter and an energy consumption meter to monitor electricity usage in the culture tanks. Aeration inside the tanks was provided through microporous hoses. Additionally, the tanks were equipped with 120 L settling units, used as needed for solids management.

Water for the culture system was prepared using clean water, disinfected with chlorine (10 mg·L^−1^) and dechlorinated through aeration. Prior to stocking, alkalinity and salinity were adjusted using sodium bicarbonate and common salt to reach 80 mg CaCO_3_·L^−1^ and 2 ppt, respectively. Then, approximately 20% of each tank’s volume was inoculated with mature biofloc water. Stocking densities ranged from 18,000 to 28,000 tilapia larvae per tank, with an average weight of ~11 mg.

During the sex reversal phase, larvae were fed diets containing 60 mg·kg^−1^ of the androgen 17-α-methyltestosterone for 26 to 32 days, following the protocol described in Section 2.4. Feed was distributed using automatic feeders, which delivered the daily amount over at least eight feedings throughout the day.

Water quality parameters—including temperature, dissolved oxygen, pH, salinity, ammonia, nitrite, alkalinity, total solids, and settleable solids—were monitored weekly during the nursery phase, according to the procedures described in Section 2.5.

At the end of each production cycle, fish were harvested to determine zootechnical performance indicators, including final weight (mg), feed conversion ratio (FCR), survival rate, productivity, sex reversal rate, and distribution across weight classes. Individuals in the smallest size class (<7.5 mm) were discarded and not included in economic performance calculations, as their growth was insufficient to ensure effective sex reversal.

To assess sex reversal success, approximately 200 individuals per batch were sampled, euthanized using deep anesthesia with eugenol (75 mg·L^−1^), and fixed in 10% buffered formalin. Histological evaluation consisted of dissection using forceps and scissors to extract the gonads, which were mounted on slides, stained with carmine acetate, and squashed under coverslips. Samples were analyzed under a light microscope (40× magnification) to determine phenotypic sex: male, intersex, or female.

For economic evaluation, the following parameters were recorded throughout the production cycle: electricity consumption, input usage (salt, sodium bicarbonate, bioremediator, alcohol, hormone, transport bags, oxygen), feed consumption, and labor. At the end of each cycle, the following indicators were calculated: effective operating cost (EOC), total operating cost (TOC), operational profit per thousand fry (OP), and net margin (NM). All values are expressed per thousand fry produced, using the methodology adopted by the Agricultural Economics and Planning Center (CEPA) of Epagri [20], and were calculated based on the following formulas:

TOC (U$)=EOC+ Asset Depreciation

Operating profit (U$)=Gross revenue−TOC

$$Net\;margin\;\left(\%\right)=\frac{Operating\;profit}{Gross\;revenue}\times 100$$

For the production cost calculations, input costs were recorded individually for each cycle (Table 1).

## 3. Results

### 3.1. Trial 1: Evaluation of Different Total Suspended Solids Management Strategies During Nile Tilapia Nursery in a Biofloc System

The lowest TSS range resulted in higher levels of dissolved oxygen, pH, and alkalinity; however, this management strategy required more frequent solid removal and exhibited higher nitrite levels (*p* < 0.05) (Table 2). Temperature remained stable at approximately 28 °C throughout the 28-day trial and did not differ between treatments (*p* > 0.05).

Regarding zootechnical performance (Table 3), the final weight and specific growth rate were significantly higher in T2 compared to T4, while treatments T1 and T3 did not differ statistically from the other treatments. Survival was not affected by the concentration of solids, remaining around 85%.

The feed conversion ratio (FCR) and final productivity were not significantly influenced by the different levels of suspended solids (*p* > 0.05), although a trend toward increased FCR was observed with higher TSS levels (T1: 1.78 ± 0.22; T4: 2.08 ± 0.11). The average final productivity of the nursery tanks in the biofloc system showed a slight decrease, from 1.62 ± 0.15 in T2 to 1.33 ± 0.07 in T4, indicating a slight reduction in yield in more loaded systems, although no statistically significant difference was observed (*p* = 0.054).

### 3.2. Trial 2: Evaluation of Stocking Density of Tilapia Larvae Cultured in a Biofloc System

Water quality parameters during the 28-day cultivation of Nile tilapia larvae in the biofloc system at different stocking densities are presented in Table 4. Water temperature, controlled by thermostat, was maintained similarly across all treatments. Likewise, water hardness and nitrate levels were not significantly influenced by the different larval stocking densities.

Dissolved oxygen values decreased with increasing stocking density, with oxygen concentrations dropping below 4 mg·L^−1^ at the end of the nursery phase in tanks stocked at 8 and 10 larvae per liter. pH and alkalinity also showed a tendency to decrease in tanks with higher stocking densities; however, mean values remained within the planned range for experimental management.

Total ammonia nitrogen levels were higher in treatments with densities above 6 larvae per liter but remained below 1 mg·L^−1^ throughout. Nitrite concentrations in culture water exceeded 1 mg·L^−1^ on average in the 8 and 10 larvae per liter treatments, reaching peaks above 2 mg·L^−1^ during cultivation.

Settleable solids (SS), total suspended solids (TSS), and the estimated amount of solids removed increased linearly with stocking density. In the highest density treatments (8 and 10 larvae per liter), difficulties in maintaining solids within desired levels were observed at the end of the nursery phase, with TSS values exceeding 700 mg·L^−1^ in these tanks.

**Figure 2:**
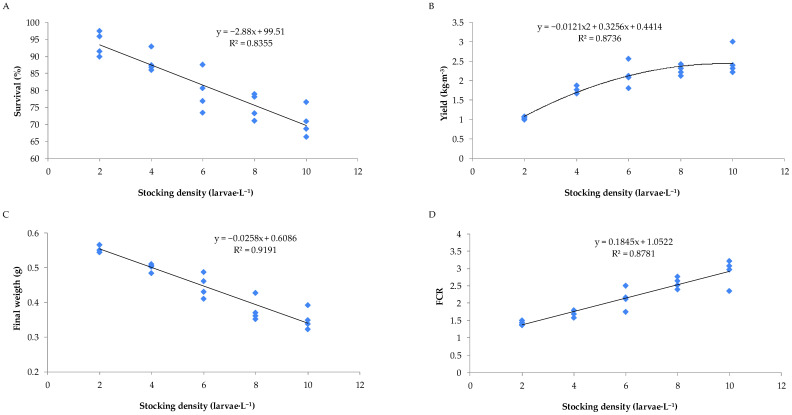
Zootechnical performance of Nile tilapia larva rearing in a bioflocs system as per different stocking densities for 28 days. (**A**) Survival; (**B**) Yield; (**C**) Final weight; (**D**) Feed conversion rate.

**Figure 3:**
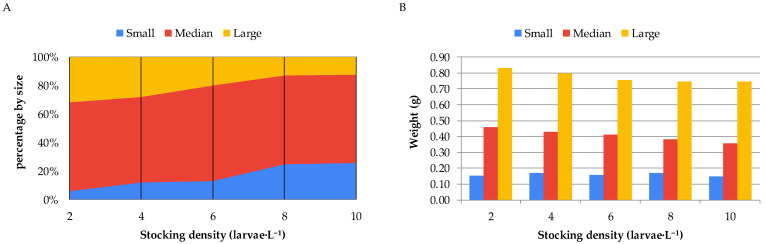
Zootechnical performance of Nile tilapia larva rearing in a bioflocs system as per different stocking densities for 28 days (Small: <7.5 mm, Median: 7.5 to 9.0 mm, Large: >9.0 mm). (**A**) Percentage distribution of fingerlings across the three size classes according to stocking density. (**B**) Mean weight of fingerlings within each size class as a function of stocking density.

The zootechnical parameters of Nile tilapia larvae during the nursery phase in the biofloc system, under different stocking densities, are presented in Figure 2 and Figure 3. Survival rates and final mean weight showed decreasing linear relationships with increasing stocking density during the biofloc nursery phase. In contrast, the feed conversion ratio exhibited a positive linear relationship with stocking density, while nursery productivity followed a quadratic trend.

When the animals reared under different stocking densities during the nursery phase of Nile tilapia in the biofloc system were classified into different size classes, it was observed that higher stocking densities resulted in a greater number of fish in the smaller size class (classified as <7.5 mm) and a lower number in the larger size class (classified as >9.0 mm). It was also observed that, within each size class, the higher the stocking density, the lower the average weight of the fry (Figure 3).

### 3.3. Trial 3: Pilot-Scale Production Monitoring

The water quality parameters monitored over the six cycles of Nile tilapia nursing in a pilot-scale biofloc system were managed according to the optimal values for the species, as determined in the previous stages, and remained within acceptable ranges for the proposed management. The periods of each cycle are described in Table 5 and occurred between November and March, which corresponds to the spring and summer seasons in the subtropical southern region of Brazil. As a result, average water temperatures during the cycles ranged from 26.9 to 28.7 °C.

Dissolved oxygen levels remained consistently above 6 mg·L^−1^, with cycle averages between 6.5 and 7.6 mg·L^−1^. The pH, which was controlled by daily additions of sodium bicarbonate, maintained average values between 7.4 and 7.7 throughout the cycles. These daily additions also kept water alkalinity within average values ranging from 68 to 115 mg·L^−1^. Total ammonia nitrogen and nitrite concentrations remained below 0.3 mg TAN·L^−1^ and 0.9 mg N-NO_2_·L^−1^, respectively, across all cycles.

Salinity was maintained between 1.5 and 2.3 ppt. Sedimentable solids (SS) and total suspended solids (TSS) showed considerable variation between culture cycles, with the lowest values observed in Cycle 5 (SS = 6 ± 5 mL·L^−1^; TSS = 226 ± 102 mg·L^−1^) and the highest in Cycle 4 (SS = 29 ± 7 mL·L^−1^; TSS = 335 ± 35 mg·L^−1^), reflecting operational particularities of each cycle. Overall, the parameters indicate that the management strategies adopted ensured favorable environmental conditions for larval development at a commercial scale.

The zootechnical parameters of Nile tilapia larvae during nursing in the pilot-scale biofloc system over six production cycles are presented in Table 5. The stocking densities used during these cycles ranged from 1.5 to 2.3 larvae per liter. The final average weight of the fry varied from 580 mg to 863 mg by the end of the nursing period. This variation was mainly due to the number of days in each nursing cycle and, to a lesser extent, to water temperature and stocking density. The average survival rates of Nile tilapia fry in the BFT ranged from 85.7% to 97.4%. Productivity during these pre-commercial scale cycles ranged from 0.9 to 1.7 kg·m^−3^.

Regarding fry classification, a small percentage of fish were observed in mesh sizes <7.5 mm. These individuals were discarded and not included in the economic analyses (Table 6).

Across the six production cycles, the proportion of small fish remained below 8%, except in cycle 5, which showed a discard rate of 18.5%. This was likely due to the use of larvae with up to 5 days of post-hatch age difference in cycle 5, whereas in the other cycles, the age difference did not exceed 2 days.

**Figure 4:**
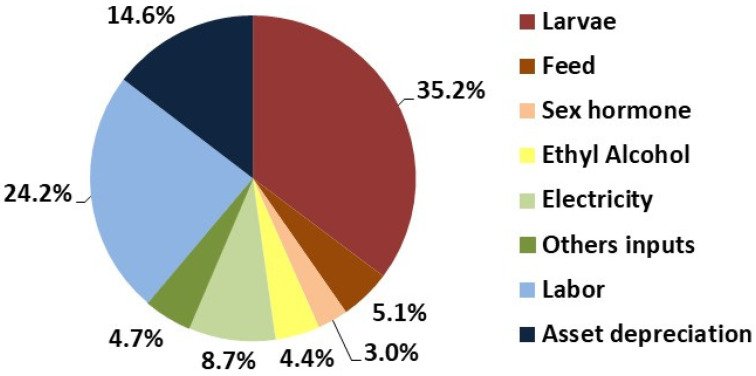
Percentage contribution of each input to the production cost of Nile tilapia fingerlings reared in biofloc systems.

The economic performance of Nile tilapia nursery production in a biofloc system is presented in Table 6 and Figure 4. Among the main production costs were larvae, labor, depreciation, and electricity, which together accounted for 82.7% of the total operational cost. The hormone-treated feed and the alcohol used for hormone application represented 12.5% of the total operational cost.

In general, the production cost of tilapia fry in the biofloc system ranged from USD 20.96 to USD 22.56 per thousand fry, except for cycle 5, which reached USD 29.36 per thousand due to the higher proportion of discarded small fish. Finally, the net profit margin for the tilapia nursery cycles in the biofloc system ranged from 37.4% to 41.9%, except for cycle 5, which was 18.5%.

## 4. Discussion

The results obtained throughout the three trials demonstrated the technical and economic potential of the biofloc system (BFT) for the Nile tilapia nursery phase, particularly when appropriate strategies are adopted for the management of suspended solids and stocking density. BFT is known for its ability to significantly reduce water exchange, benefiting both the production system and the environment. However, production success is directly linked to proper water quality management [8].

In the first and second trials, lower levels of total suspended solids (TSS) and lower stocking densities resulted in better water quality conditions. Water quality parameters must be controlled to remain within optimal ranges for the species, as imbalances can negatively affect the growth of aquatic species and the functionality of the bacterial community [8]. When kept within optimal ranges, these parameters reflect a more stable and favorable environment for larval development, corroborating previous studies that emphasize the importance of managing solids and stocking density for BFT system stability and fish performance [7,8,9,21]. Furthermore, lower TSS ranges (200–400 mg·L^−1^) resulted in higher final weights and specific growth rates. Several studies support these findings, demonstrating that lower TSS values (ideal range between 200 and 500 mg·L^−1^) are more suitable for aquatic animals. TSS plays a crucial role in water quality, especially regarding nitrogen cycling and avoiding competition for dissolved oxygen and micronutrients among bacteria [8,22,23,24,25]. Excessive TSS can hinder growth and cause significant gill obstruction [26]. Maintaining TSS at ideal levels enhances BFT performance by balancing bacterial populations and system productivity [25,27,28].

Another important aspect related to TSS, and directly associated with the third trial, is the process of hormonal sex reversal. It is well known that successful sex reversal requires consistent ingestion of feed containing masculinizing hormones [10,15,29]. However, BFT also serves as a natural food source for fish [7,10], raising concerns that elevated TSS levels might lead to inadequate hormone intake and, therefore, unsuccessful sex reversal. Nonetheless, when hormone feed was administered under low-TSS conditions, high sex reversal rates were observed [11].

Regarding water quality parameters, temperature was stable across treatments in the first two trials, as it was controlled by thermostats (28 °C). Dissolved oxygen showed a slight decrease as TSS increased, with significantly lower levels in T4 compared to T1 and T2, though levels remained adequate for larval development [30]. Nitrite concentrations reached potentially toxic levels (>3 mg·L^−1^) at the end of the culture in treatments with the lowest TSS. Similar results were reported in marine shrimp (*Litopenaeus vannamei*) cultures in BFT systems with TSS below 200 mg·L^−1^, where nitrifying bacterial populations were insufficient to manage the nitrogen load [24]. However, these levels did not impair larval performance in the current study due to the use of slightly saline water (~2 ppt), as chloride ions from salt reduce nitrite toxicity by competing for uptake at the gill level [31].

The second trial revealed that stocking density is a critical factor in the success of tilapia nursery in BFT. Higher densities led to solids accumulation, elevated nitrogenous compounds, and reduced dissolved oxygen, which negatively affected zootechnical performance, particularly survival and final weight. These findings align with the literature that stresses the importance of balancing biomass load with the system’s ability to maintain water quality [8,9,32]. Higher stocking densities also promoted an increase in TSS. This increase may have contributed to the higher mortality observed at greater densities, since TSS accumulation can be harmful to aquatic organisms [8,11]. Despite increased productivity at higher densities, elevated feed conversion ratios and lower size uniformity suggest potential biological and operational limitations, especially in the absence of additional biofiltration or partial water exchange.

In the third, pilot-scale trial, the system’s feasibility under near-commercial conditions was validated. High survival rates (>85%) and adequate zootechnical performance were observed at moderate stocking densities. Sex reversal efficiency reached a minimum of 99.6%, with a maximum of 0.4% intersex individuals and no females detected. According to Silva et al. [14], 25 days of nursery in earthen ponds with 17α-methyltestosterone feed are sufficient for effective sex reversal. This aligns with the current study, which achieved 99.8% reversal and only 0.2% intersex in 26 days under BFT conditions. Based on these findings, an optimized approach for sex reversal in super-intensive systems may involve initially stocking larvae at higher densities (6–10 larvae·L^−1^) during the first 7–10 days, followed by thinning to 1.5–2.5 larvae·L^−1^.

Although operational costs varied among cycles, the net margins were positive in all cases, confirming the economic feasibility of BFT for tilapia fry production. These results are particularly relevant in the current aquaculture scenario, which seeks to combine sustainability and profitability. However, to date, no literature has provided detailed financial analyses of this production phase and sex reversal process for Nile tilapia in BFT systems.

BFT is considered more environmentally sustainable than conventional systems, as it maximizes production by balancing microbial and aquatic communities [9,33]. In terms of water usage, BFT significantly reduces water consumption. According to Jatobá et al. [7], producing one ton of Nile tilapia requires nearly 200 times more water in conventional systems than in BFT, potentially leading to water savings of up to 30% [34]. Some authors, however, note the system’s high energy demand, which may compromise its sustainability [35,36]. In our study, electricity accounted for only 8.7% of the total operational cost of fry production in BFT. Thus, the cost of production reported here is competitive with that of earthen pond systems, particularly due to the higher survival rates achieved in BFT [12]. Increased survival can dilute operational costs, resulting in significant economic savings.

Cost reduction is crucial in aquaculture but must be balanced with profitability [37]. Reported profit margins and returns on investment vary widely, from 8% to 20% and 45% to 57%, respectively [38,39,40,41], highlighting the influence of production costs and market prices. Nonetheless, Bezerra et al. [42], in a risk and economic feasibility analysis of Nile tilapia in BFT, reported positive profit margins in most scenarios, with 87.29% of simulations yielding profit. They concluded that juvenile production in BFT is economically viable. In the present study, all nursery cycles over two years showed net margins above 18%.

Despite these promising results, the success of BFT depends on careful management, particularly regarding organic load, feeding rates, and solids control [9]. Its economic success also depends on specific technical and market conditions, reinforcing the need for tailored management and planning [42]. Future research should explore the use of automated tools for continuous water quality monitoring and the development of more specific nutritional strategies for super-intensive systems, aiming to further enhance performance and reduce costs.

## 5. Conclusions

The BFT proved to be a viable and sustainable alternative for the Nile tilapia nursery phase, especially when appropriate strategies for managing TSS and stocking density are implemented. Low TSS levels (200–600 mg·L^−1^) and moderate stocking densities (2–6 larvae·L^−1^) promoted water quality, zootechnical performance, and fry survival, while ensuring high sex reversal rates. Economic analysis confirms the profitability of BFT under pilot-scale conditions (37.4% to 41.9%). Therefore, BFT shows strong potential to support environmentally responsible aquaculture.

## Figures and Tables

**Table 1:** Costs of the inputs used in the calculation of the effective operational costs for Nile tilapia fingerlings reared in biofloc systems.

Items	Unit	Unitary Value (U$)
Larvae	thousand	6.87
Powder feed (52%CP)	kg	1.20
Ethyl alcohol 96GL ^1^	L	2.61
Sex Hormone MT ^2^	g	12.02
Electricity (kWh) ^3^	U$·kWh^−1^	0.11
Sodium bicarbonate	kg	1.34
Probiotic ^4^	kg	29.18
Commun salt	kg	0.24
Labor (tank preparation) ^5^	MD ^12^	28.28
Labor (tank stocking) ^6^	MD ^12^	28.28
Labor (feeding) ^7^	MD ^12^	28.28
Labor (decanter handling) ^8^	MD ^12^	28.28
Labor (fish biometrics) ^8^	MD ^12^	28.28
Labor (input application) ^8^	MD ^12^	28.28
Labor (harvesting) ^9^	MD ^12^	28.28
Fish transport bags ^10^	un.	0.17
Oxygen ^11^	m^3^	2.39

^1^ Used at a ratio of 400 mL per kg of feed. ^2^ Used at 60 mg per kg of feed. ^3^ Total energy consumption of the greenhouse was measured and apportioned based on the ratio of hose length installed in the production units to the total hose length installed in the greenhouse. ^4^ Used at 5 g per week per production unit. ^5^ Labor cost considered for one worker for 2 h (0.250 workday equivalents). ^6^ Labor cost considered per production cycle for one worker for 1 h (0.125 workday equivalents). ^7^ Labor cost considered per day of culture for one worker for 30 min (0.0625 workday equivalents). ^8^ Labor cost considered per management day during the culture period for one worker for 30 min (0.0625 workday equivalents). ^9^ Labor cost considered per cycle for two workers for 4 h (1.00 workday equivalent). ^10^ One unit used per thousand fish sold. ^11^ 0.04 m^3^ used per fish transport bag. ^12^ Man-day—Average value of rural salaried employees in Santa Catarina in Abril 2025 [20].

**Table 2:** Water parameters of Nile tilapia larva rearing in a bioflocs system as per different solids management for 28 days.

Water Parameters	T1	T2	T3	T4	SEM	*p*-Value
Temperature (°C)	28.76	28.49	28.69	28.73	0.045	0.115
Dissolved oxygen (mg·L^−1^)	6.92 ^a^	6.96 ^a^	6.1 ^ab^	6.70 ^b^	0.030	0.001
TAN (mg·L^−1^)	0.22	0.20	0.22	0.25	0.009	0.216
N-Nitrite (mg·L^−1^)	1.06 ^a^	0.50 ^b^	0.60 ^b^	0.56 ^b^	0.062	0.001
N-Nitrate (mg·L^−1^)	196.00	211.08	212.08	206.17	4.905	0.685
pH	7.93 ^a^	7.88 ^a^	7.80 ^b^	7.75 ^b^	0.019	0.001
Alkalinity (mg CaCO_3_·L^−1^)	84.19 ^a^	80.94 ^ab^	76.92 ^bc^	74.33 ^c^	1.172	0.002
Hardness (mg·L^−1^)	170.88	177.13	181.00	186.38	2.175	0.059
SS (mL·L^−1^)	6.61 ^d^	13.69 ^c^	27.17 ^b^	41.25 ^a^	3.440	0.001
TSS (mg·L^−1^)	179.43 ^d^	298.06 ^c^	466.63 ^b^	631.10 ^a^	44.169	0.001
Estimated solids removed (g·tank^−1^)	73.83 ^a^	36.96 ^b^	25.69 ^c^	16.94 ^d^	5.654	0.001

T1: 100–200 mg·L^−1^. T2: 200–400 mg·L^−1^. T3: 400–600 mg·L^−1^. T4: 600–800 mg·L^−1^. SEM: Standard Error of Mean. TAN: total ammonia nitrogen. SS: Settleable solids. TSS: Total suspended solids. Data are expressed as mean. Different letters represent statistically significant differences (*p* < 0.05) between treatments in Tukey’s test.

**Table 3:** Zootechnical parameters of Nile tilapia larva rearing in a bioflocs system as per different solids management for 28 days.

Parameters	T1	T2	T3	T4	SEM	*p*-Value
Final weight (g)	0.61 ^ab^	0.62 ^a^	0.54 ^ab^	0.52 ^b^	0.015	0.016
Survival (%)	84.75	86.58	85.08	85.58	0.989	0.937
FCR	1.78	1.71	2.03	2.08	0.062	0.069
SGR (%)	6.33 ^ab^	6.34 ^a^	6.20 ^ab^	6.16 ^b^	0.028	0.018
Yield (kg·m^−3^)	1.56	1.62	1.38	1.33	0.046	0.054

T1: 100–200 mg·L^−1^. T2: 200–400 mg·L^−1^. T3: 400–600 mg·L^−1^. T4: 600–800 mg·L^−1^. SEM: Standard Error of Mean. FCR: Feed Conversion Ratio. SGR: Specific Growth Rate. Data are expressed as mean. Different letters represent statistically significant differences (*p* < 0.05) between treatments in Tukey’s test.

**Table 4:** Water parameters of Nile tilapia larva rearing in a bioflocs system as per different stocking densities for 28 days.

Water Parameters	Stocking Density (Larvae·L^−1^)					SEM	*p*-Value
	2	4	6	8	10		
Temperature (°C)	28.85	28.88	28.69	28.64	28.74	0.035	0.112
Dissolved oxygen (mg·L^−1^)	6.82 ^a^	6.52 ^ab^	6.26 ^b^	5.79 ^c^	5.69 ^c^	0.106	0.001
TAN (mg·L^−1^)	0.15 ^b^	0.16 ^b^	0.24 ^ab^	0.35 ^a^	0.35 ^a^	0.024	0.01
N-Nitrite (mg·L^−1^)	0.40 ^c^	0.65 ^bc^	0.64 ^bc^	1.52 ^ab^	1.12 ^a^	0.108	0.001
N-Nitrate (mg·L^−1^)	90.42	87.92	103.42	111.58	95.83	3.958	0.323
pH	8.09 ^a^	7.79 ^b^	7.69 ^bc^	7.63 ^c^	7.73 ^bc^	0.039	0.001
Alkalinity (mg·CaCO_3_·L^−1^)	102.86 ^a^	89.57 ^b^	79.75 ^bc^	76.43 ^c^	84.64 ^bc^	2.367	0.001
Hardness (mg·L^−1^)	77.50	73.25	74.25	74.25	75.00	0.599	0.219
SS (mL·L^−1^)	11.84 ^c^	13.58 ^bc^	15.50 ^ab^	16.45 ^a^	18.06 ^a^	0.563	0.001
TSS (mg·L^−1^)	250.56 ^e^	311.44 ^d^	367.25 ^c^	419.31 ^b^	450.69 ^a^	16.812	0.001
Estimated solids removed ^1^ (g·tank^−1^)	-	24.25 ^d^	55.81 ^c^	123.03 ^b^	144.80 ^a^	12.904	0.001

SEM: Standard Error of Mean. TAN: total ammonia nitrogen. SS: Settleable solids. TSS: Total suspended solids. Data are expressed as mean. Different letters represent statistically significant differences (*p* < 0.05) between treatments in Tukey’s test. ^1^: At a density of 2 larvae·L^−1^, solid removal was not required throughout the experiment. At densities of 4 and 6 larvae·L^−1^, 3 and 6 solid removal events were performed, respectively. For higher densities, namely 8 and 10 larvae·L^−1^, 7 solid removal interventions were necessary.

**Table 5:** Fish growth performance of Nile tilapia hatchery reared in biofloc systems.

Parameters	Rearing 1	Rearing 2	Rearing 3	Rearing 4	Rearing 5	Rearing 6
Stocking date	11/09/2023	11/24/2023	12/18/2023	12/18/2023	02/24/2024	12/18/2024
Harvesting date	12/07/2023	12/27/2023	01/22/2024	01/22/2024	03/26/2024	01/13/2025
Larvae stored	22,000	28,000	26,500	22,980	18,000	20,000
Density (larvae·L^−1^)	1.8	2.3	2.2	1.9	1.5	1.7
Final weight (mg)	580	743	814	772	863	581
Feed conversion rate	1.37	1.26	1.32	1.05	1.24	1.19
Final productivity (Kg·m^−3^)	0.93	1.48	1.69	1.44	1.22	0.91
Survival (%)	87.7	85.7	94.0	97.4	94.5	93.5
Sex reversal rate (% males)	100.0	100.0	99.6	99.6	100.0	99.8
Sex reversal rate (% intersex)	0.0	0.0	0.4	0.4	0.0	0.2
Sex reversal rate (% female)	0.0	0.0	0.0	0.0	0.0	0.0
Size classification (<7.5 mm)	0.5	4.5	7.0	4.8 ^1^	18.5	7.8
Size classification (7.5 to 9.0 mm)	57.0	51.1	47.5	53.9 ^1^	77.6	73.1
Size classification (>9.0 mm)	42.5	44.4	45.5	41.3 ^1^	3.9	19.1
Time (days)	28	29	32	32	31	26

^1^ Classified in sizes of <8.0 m, 8.0 to 10.0 mm and >10.0 mm.

**Table 6:** Production cost per thousand of Nile tilapia fingerlings reared in biofloc systems.

Item	Value per Thousand (US$ thousand^−1^)					
	Rearing 1	Rearing 2	Rearing 3	Rearing 4	Rearing 5	Rearing 6
Larvae	7.87	8.40	7.85	7.41	8.92	7.97
Feed	0.95	1.13	1.39	1.02	1.58	0.90
Sex hormone MT ^1^	0.57	0.68	0.83	0.61	0.95	0.54
Ethyl alcohol (96GL)	0.83	0.98	1.20	0.89	1.37	0.78
Electricity	1.86	1.61	1.76	1.91	2.85	1.92
Other inputs ^2^	1.01	1.01	1.09	0.99	1.35	1.03
Labor	5.34	4.55	4.73	5.14	7.78	5.74
EOC	18.43	18.35	18.84	17.98	24.78	18.88
TOC	21.74	21.12	21.58	20.96	29.36	22.56
Gross revenue	36.05	36.05	36.05	36.05	36.05	36.05
Operating profit	14.31	14.93	14.47	15.09	6.69	13.49
Net margin (%)	39.7%	41.4%	40.1%	41.9%	18.5%	37.4%

^1^ 17-α-methyltestosterone. ^2^ Sodium bicarbonate, common salt, probiotic, pure oxygen, fish transport bags. EOC: Effective operational cost. TOC: Total operational cost. Values quoted in reais (R$) and converted into dollars (US$ 1.00 = R$ 5.825) in April 2025 according to the Central Bank of Brazil.

## Data Availability

The data presented in this study are available on request from the corresponding author.

## Abbreviations

The following abbreviations are used in this manuscript:

BFT	Biofloc Technology System
CEPA	Agricultural Economics and Planning Center
CEPIT	Itajaí Experimental Aquaculture Station
CEUA	Animal Ethics Committee
DO	Dissolved Oxygen
EOC	Effective Operating Cost
EPAGRI	Agricultural Research and Rural Extension Company Of Santa Catarina
FCR	Feed Conversion Ratio
NM	Net Margin
OP	Operational Profit
SGR	Specific Growth Rate
SS	Settleable Solids
TAN	Total Ammonia Nitrogen
TOC	Total Operating Cost
TSS	Total Suspended Solids
VSS	Volume Of Settleable Solids
